# Chloroform Anæsthesia without Danger

**Published:** 1894-10

**Authors:** Frank H. Pritchard


### Chloroform Anæsthesia without Danger.

BY FRANK H. PRITCHARD, M.D.

Dr. G. Maurange (*La Semaine Medicale*, No. 27, 1894) recommends the subcutaneous injection of the following solution as a preparatory and prophylactic measure before administering chloroform:

| | |
|---|---|
| Neutral sulphate sparteine (grs. ivss). | 0.30 |
| Muriate morphine (grs. jss). | 0.10 |
| Sterilized water, ad (ʒijss) | 10 ccms. |

Inject 1 ccm. of this solution a quarter of an hour before administering the anæsthetic.

Out of 106 operations where chloroform was employed with preliminary injection of this cardiac stimulant, not a single disagreeable symptom was observed, however long the subject was under the anæsthetic. The period of excitement was suppressed and the absorption of the anæsthetic was reduced to a minimum on account of the morphine.

(The employment of a preliminary injection of atropine with morphine has been known for quite awhile. I have not much confidence in atropine as a heart stimulant, and believe that its use is limited to a peculiar state of the heart, but would rather confide in nitro-glycerine with or without digitaline or digitalis, sparteine or strophanthus, in the prevention of disagreeable accidents in chloroform anæsthesia, as well as that of cocaine. I have full confidence in nitro-glycerine as a rapid heart stimulant, either alone or combined with digitalis. A recent German writer, in writing on the physiological antagonism of drugs, states that digitalis is the specific antidote for chloroform. He begins a day or so before the operation and administers the drug so that the system be well under its influence, and has by this method succeeded in doing a large number of operations under chloroform without the slightest accident. Sparteine also has the advantage of acting quite rapidly, yet not so quickly as nitro-glycerine or amyl nitrite—another antidote for heart failure under chloroform. In painless extraction of teeth I have found the addition of a few drops of an alcoholic solution (1:100) of the drug to a ½ per cent. solution of cocaine with carbolic acid to give the best of results, yet with the same preparation without the nitro-glycerine I have in at least half the cases observed either slight or pronounced symptoms of cocaine intoxication. Not too much of the stimulant is to be added, or the effect of the cocaine will be more or less antidoted and the anæsthesia will be imperfect or lacking. The formula given in a recent number of the *Lancet-Clinic* I have found to contain too much nitro-glycerine. Five to ten drops to the ounce are sufficient, in my opinion. The technique also requires careful study. It is dangerous to use the secret preparations, for no one knows how much cocaine they may contain; some persons have a curious idea of the lack of power of the drug. —TRANS.)—*Cincinnati Lancet-Clinic.*

